# Noninvasive Peroneal Sensory and Motor Nerve Conduction Recordings in the Rabbit Distal Hindlimb: Feasibility, Variability and Neuropathy Measure

**DOI:** 10.1371/journal.pone.0092694

**Published:** 2014-03-21

**Authors:** John R. Hotson

**Affiliations:** 1 Department of Neurology and Neurological Sciences, Stanford University, Stanford, California, United States of America; 2 California Institute for Medical Research, San Jose, California, United States of America; University of Florida, United States of America

## Abstract

The peroneal nerve anatomy of the rabbit distal hindlimb is similar to humans, but reports of distal peroneal nerve conduction studies were not identified with a literature search. Distal sensorimotor recordings may be useful for studying rabbit models of length-dependent peripheral neuropathy. Surface electrodes were adhered to the dorsal rabbit foot overlying the extensor digitorum brevis muscle and the superficial peroneal nerve. The deep and superficial peroneal nerves were stimulated above the ankle and the common peroneal nerve was stimulated at the knee. The nerve conduction studies were repeated twice with a one-week intertest interval to determine measurement variability. Intravenous vincristine was used to produce a peripheral neuropathy. Repeat recordings measured the response to vincristine. A compound muscle action potential and a sensory nerve action potential were evoked in all rabbits. The compound muscle action potential mean amplitude was 0.29 mV (SD ± 0.12) and the fibula head to ankle mean motor conduction velocity was 46.5 m/s (SD ± 2.9). The sensory nerve action potential mean amplitude was 22.8 μV (SD ± 2.8) and the distal sensory conduction velocity was 38.8 m/s (SD ± 2.2). Sensorimotor latencies and velocities were least variable between two test sessions (coefficient of variation  =  2.6–5.9%), sensory potential amplitudes were intermediate (coefficient of variation  =  11.1%) and compound potential amplitudes were the most variable (coefficient of variation  = 19.3%). Vincristine abolished compound muscle action potentials and reduced sensory nerve action potential amplitudes by 42–57% while having little effect on velocity. Rabbit distal hindlimb nerve conduction studies are feasible with surface recordings and stimulation. The evoked distal sensory potentials have amplitudes, configurations and recording techniques that are similar to humans and may be valuable for measuring large sensory fiber function in chronic models of peripheral neuropathies.

## Introduction

The peroneal nerve anatomy of the rabbit distal hindlimb is similar to humans. The superficial peroneal nerve widely branches to innervate the dorsal skin of the rabbit foot. The proximal dorsum of the foot also contains an extensor digitorum brevis muscle that is innervated by the deep peroneal nerve. The superficial peroneal nerve traverses the extensor digitorum brevis as it begins its branching. The superficial and deep peroneal nerves divide from the common peroneal nerve below the fibula head [1].

Stimulation of the rabbit common peroneal nerve at the fibula head evokes a compound muscle action potential (CMAP) from the tibialis anterior that has been used for motor unit number estimation [2]. Stimulation of the rabbit sciatic and tibial nerve evokes a CMAP from foot plantar muscles and has been used to measure motor conduction in a nerve compression model [3], [4] and in a rabbit model of Guillian-Barré syndrome [5], [6]. The rabbit foot anatomy suggested that stimulating the distal deep and superficial peroneal nerves could evoke a CMAP and a sensory nerve action potential (SNAP) respectively. Reports of such distal peroneal nerve conduction studies in the rabbit, however, were not identified with a literature search. The possibility of recording a SNAP was of particular interest because a reduction of SNAP amplitude may be a prominent marker of a large-fiber, length-dependent sensory neuropathy [7]–[9]. Therefore, the feasibility of measuring distal sensory and motor potentials from the rabbit's foot was determined and it proved to be practical.

A subsequent aim was to show the potential use of the distal peroneal nerve conduction methods in measuring the time course of a peripheral nerve disorder. A neuropathy was induced by the chemotherapy drug vincristine, a neurotoxin that produces a sensorimotor neuropathy in both rabbits [10]–[12] and humans [13]–[15].

## Materials and Methods

### Ethics statement

This study was carried out in accordance with the recommendations in the Guide for the Care and Use of Laboratory Animals of the National Institutes of Health. The protocol was approved by the Institutional Animal Care and Use Committee of the California Institute for Medical Research (Permit # 11–2.2).

### Nerve conduction measurements

Six female New Zealand white rabbits weighing 4–5 kg were tested. The rabbits had previously been used in an approved protocol that asked if an axon transport inhibitor delayed recovery from botulinum toxin neuroblockade. This preceding study injected botulinum toxin and vincristine into a unilateral tibialis anterior and measured the response of the muscle's CMAP [16]. In the current study CMAPs and SNAPs were recorded from the limb contralateral to the earlier injected leg beginning 3 months after recovery from the preceding experiment. The previously studied rabbits were used because they were available and seemed suitable to test new methodology.

**Figure 1:**
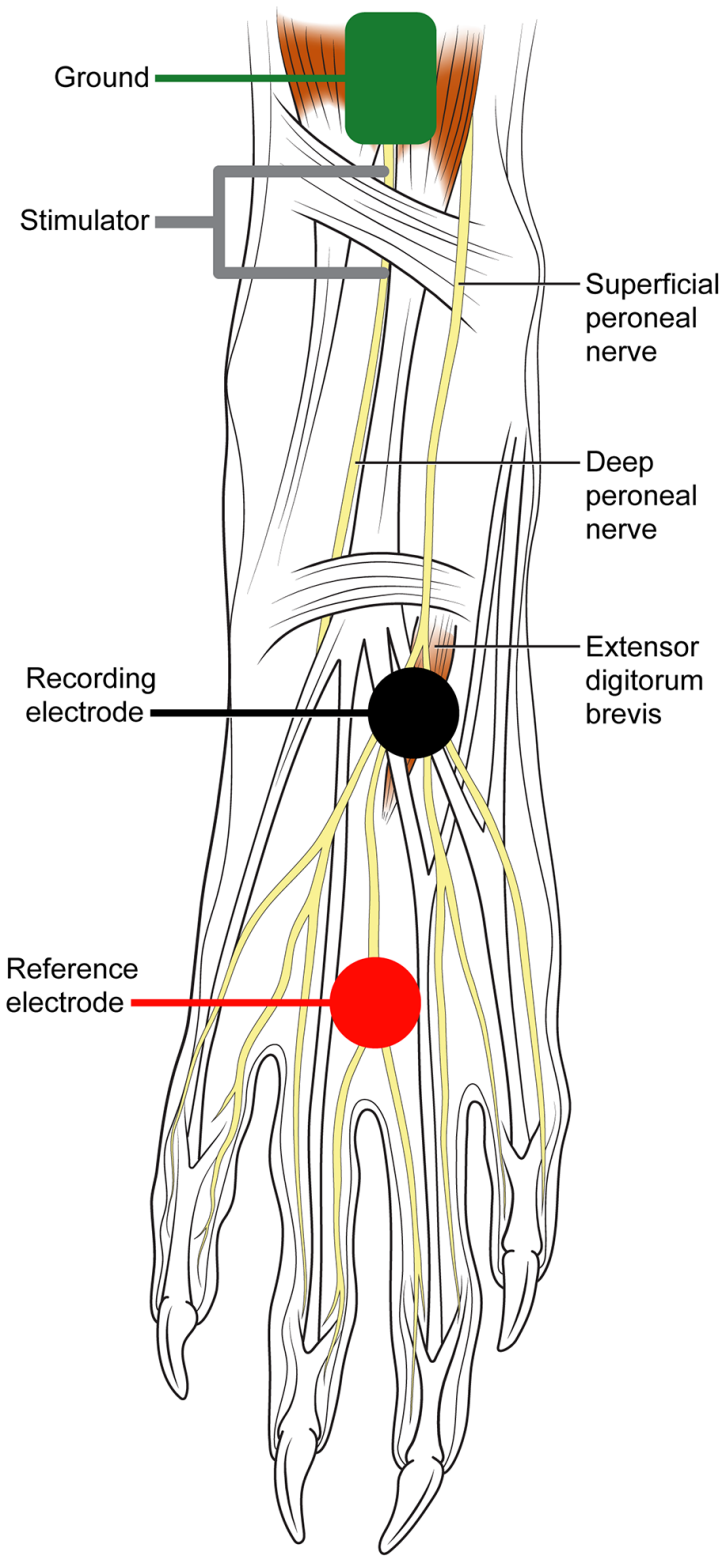
Schematic of the experimental setup and the distal rabbit hindlimb. The deep peroneal nerve was medially located and the superficial peroneal cutaneous nerve was laterally positioned (personal observation) [1]. Only a single recording site overlying both the extensor digitorum brevis and the cutaneous nerve was needed to record a CMAP and a SNAP. A CMAP was evoked by stimulating the deep peroneal nerve 4 cm proximal to the recording electrode and the common peroneal nerve at the fibular head. A SNAP was evoked by stimulating the superficial peroneal nerve 4 cm proximal to the recording electrode. The distal nerve locations facilitated evoking a SNAP uncontaminated by the larger CMAP. Illustration by Julia Stack, www.drawbones.com.

Rabbits were anesthetized with subcutaneous ketamine 35 mg/kg, xylazine 5 mg/kg and acepromazine 0.75 mg/kg during nerve conduction studies. The hair from the rabbit's leg and dorsal foot was removed with clippers and a depilatory cream. A 20 mm circular adhesive recording electrode (CareFusion #415000) was attached to the proximal dorsal midline of the foot and a reference electrode was placed 3 cm distal (Fig.1). Both the extensor digitorum brevis CMAP and the superficial peroneal SNAP were recorded from the same electrode site. A 20 mm circular ground electrode was attached to the lateral foot proximal to the recording electrode (Fig. 1). Recordings were obtained with an Excel Neuromax 2008 electromyography machine.

The peroneal nerve was stimulated with a surface stimulator (Cadwell #302151) with 1.3 cm spacing between the tips of the adjustable cathode and anode. CMAPs were evoked by stimulating the deep peroneal nerve 4 cm proximal to the recording electrode just medial to midline and the common peroneal nerve at the fibular head. A SNAP was evoked by stimulating the superficial peroneal nerve 4 cm proximal to the recording electrode, lateral to the midline. The lateral position of the superficial peroneal nerve allowed it to be stimulated in isolation without spread of current to the deep peroneal nerve (Fig. 1). The study did not attempt to record a sensory potential with proximal stimulation of the peroneal nerve at the knee nor did it attempt to stimulate the superficial peroneal where it divides from the common peroneal nerve using near-nerve needle electrodes. The stimulus was a 0.1 ms square wave current pulse, with an intensity 10% supramaximal. Hindlimb temperature was maintained above 32°C with a heating pad and heat lamp.

CMAP and SNAP latencies were measured to the onset of the initial negative deflection. SNAP negative peak latencies were also measured. The peroneal motor conduction velocity was calculated by dividing the distance between distal and proximal stimulation sites by the corresponding latency difference. Sensory velocity was the 4 cm distance between the stimulating and recording sites divided by the onset distal latency. The CMAP and SNAP amplitudes were measured from onset of the initial negative deflection to the negative peak. SNAP measurements were obtained for single nerve stimulation and from the average of 3 SNAPs. The sensory and motor measures from two separate test sessions were averaged for each rabbit.

In one rabbit 1 ml of lidocaine 0.5% (5 mg/ml) was injected at the motor and sensory nerve stimulation sites above the ankle and 1 ml was injected just distal to these sites. Only one rabbit was tested in order to keep the total number of test sessions to a minimum.

### Intertest variability

In order to evaluate the intertest variability of the peroneal CMAP and SNAP, the six rabbits were tested and retested with a one-week interval. The distance from the first toe proximal joint to the recording electrode was measured and kept constant over the repeated test sessions. Variability was expressed by the mean percent difference between test-retest values and by the coefficient of variation statistic. The mean percent difference was calculated from the absolute differences between test-retest values divided by their mean values [2]. The coefficient of variation equaled the within-subject variation divided by the mean of the test-retest differences and was expressed as a percentage. The within-subject variation or standard error was the standard deviation of the test-retest differences divided by √2 [17].

### Neuropathy measure

Three rabbits were available for a repeated-measures study of peripheral nerve dysfunction produced by vincristine. Distal hindlimb sensorimotor recordings were obtained as described above except the ground electrode was changed to a 20×25 mm rectangular electrode (CareFusion # 406600) placed on the anterior tibialis immediately proximal to the surface stimulator anode site. Skin preparation was easier over the anterior tibialis and there was no difference in background noise between the new ground site and lateral foot site.

Past rabbit studies of vincristine neuropathy have given intravenous vincristine 0.2 mg/kg or higher weekly for 5 weeks to produce pathologic evidence of toxic nerve damage [10]–[12]. Vincristine 0.1 mg/kg weekly for five weeks reportedly does not cause gross pathology in peripheral nerves. Therefore two rabbits received weekly intravenous vincristine 0.2 mg/kg via a marginal ear vein. A single control rabbit received intravenous saline.

Baseline nerve conduction studies were obtained prior to vincristine injections. The same studies were performed biweekly after the vincristine injection for 4 weeks and then weekly. The percent post-vincristine/baseline pre-vincristine values were calculated for the repeated recordings. The low number of rabbits in this feasibility study precluded a meaningful statistical analysis.

## Results

### Nerve conduction measurements

**Figure 2:**
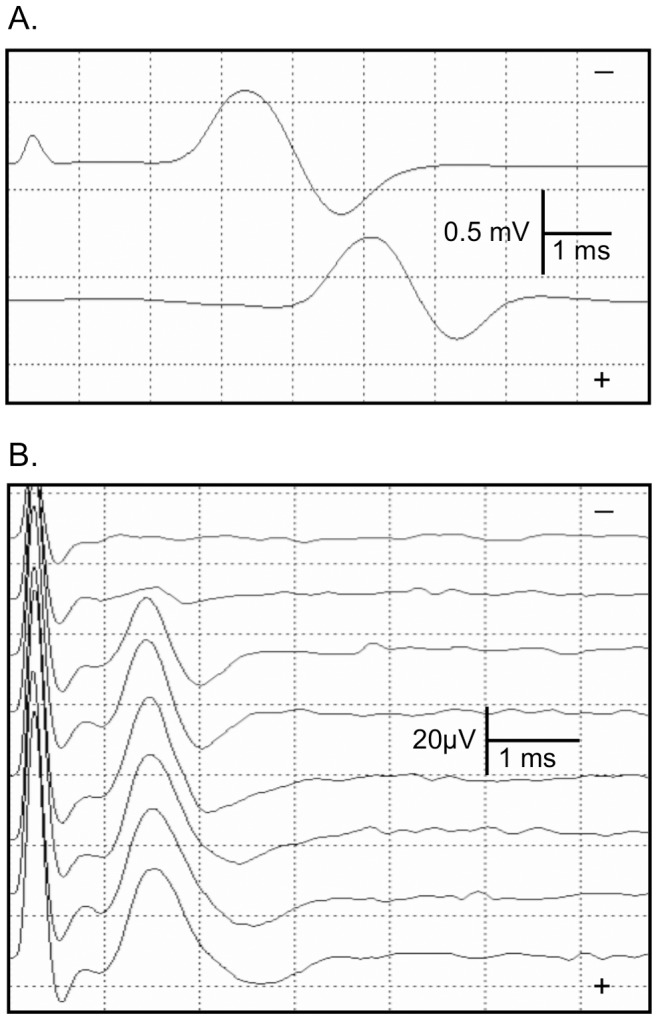
CMAP and SNAP recordings. A. CMAP evoked by distal (top trace) and proximal (bottom trace) stimulation. B. SNAP stimulus-response curve with 1 mA stimulus step increments starting subthreshold at the top trace and increasing to supramaximal by the bottom trace.

Stimulation of the deep peroneal and superficial peroneal nerves evoked CMAPs and SNAPs, respectively, in all six rabbits (Fig. 2). The CMAP was evoked with the lowest threshold just medial of the anterior midline of the distal leg, while the SNAP was evoked lateral to midline consistent with anatomic location of the deep and superficial peroneal nerves (Fig. 1) [1]. Stimulation at the ankle evoked a CMAP with an initial negative waveform. With stimulation at the fibula head, a shallow, slow positive potential commonly preceded the CMAP negative waveform. This positive potential probably reflects remote spread of a far-field potential from peroneal innervated muscles in the anterior leg. Such an initial positivity also occurs in humans with increased amplification of the extensor digitorum brevis CMAP. Table 1 summarizes the sensorimotor conduction measurements for all 6 rabbits.

**Table 1:** Nerve conduction measurements.

Nerve	Amplitude	Distal Latency	Peak Latency	Velocity
Motor	0.29±0.12 mV	3.0 ±0.3 ms	**	46.5±2.9 m/s
Sensory	22.8±2.8 μV	1.0±0.1 ms	1.6±0.1 ms	38.8±2.2 m/s
Sensory (average)	22.5±2.8 μV	1.0±0.1 ms	1.6±0.1 ms	39.1±2.1 m/s

Means ± one standard deviations are presented in Tables 1 & 2.

SNAP measurements were obtained for single nerve stimulation and from the average of 3 SNAPs (Tables 1–3).

**Motor peak latency was not measured

Stimulus-response curves showed a progressive increase in CMAP and SNAP amplitude as stimulus intensity was increased by small step increments in all rabbits (Fig. 2). The surface stimulation site 4 cm proximal to the recording electrode reliably evoked a supramaximal SNAP with a clear onset. Moving the stimulator cathode 1.5 cm from proximal to distal ankle sites decreased CMAP distal latency and SNAP peak latency 0.2–0.3 ms. SNAP onset became obscured by a stimulus artifact at the more distal site.

In one rabbit lidocaine 0.5% was infiltrated along the superficial peroneal and deep peroneal nerves above the ankle. Both the CMAP and SNAP were completely blocked 5 minutes after the lidocaine infiltration. The CMAP reappeared at 70 minutes after lidocaine, while the SNAP reappeared 100 minutes after the injection.

The above findings indicate that non-invasive peroneal sensorimotor nerve conduction studies in the distal hindlimb of the rabbit are feasible. The evoked sensory potentials are robust.

### Intertest variability

The peroneal nerve conduction studies were repeated twice with a one-week intertest interval in order to determine measurement variability. The mean percent difference and the coefficient of variation were lowest for the motor and sensory latency and velocity repeated measures (Table 2 & 3). The variability of the SNAP amplitude was intermediate. The CMAP amplitude had the greatest difference between test and retest measurements. Small 1–2 mm lateral or medial shifts in the recording electrode site produced relatively large changes in the CMAP amplitude. Subsequent anatomic examination of the extensor digitorum brevis revealed a narrow, cone-shaped muscle that tapered at the distal end. This muscle configuration may contribute to the variability of the CMAP amplitude.

**Table 2:** Mean percent differences between test and retest measurements.

Nerve	Amplitude	Distal Latency	Peak Latency	Velocity
Motor	24.1%±17.0	6.8%±4.2	**	2.9%±3.0
Sensory	12.1%±9.5	6.2%±7.5	3.2%±3.5	6.2%±7.5
Sensory (average)	13.6%±6.7	6.2%±7.5	5.2%±2.6	4.6%±7.7

**Motor peak latency was not measured.

**Table 3:** Coefficient of Variation.

Nerve	Amplitude	Distal Latency	Peak Latency	Velocity
Motor	19.3%	5.9%	**	2.6%
Sensory	11.1%	5.6%	3.3%	5.1%
Sensory (average)	10.4%	5.6%	3.7%	5.1%

Coefficient of Variation  =  ((SD of the test-retest differences/√2)/mean

of the test-retest differences) expressed as a percentage value.

**Motor peak latency was not measured.

### Neuropathy measure

**Figure 3:**
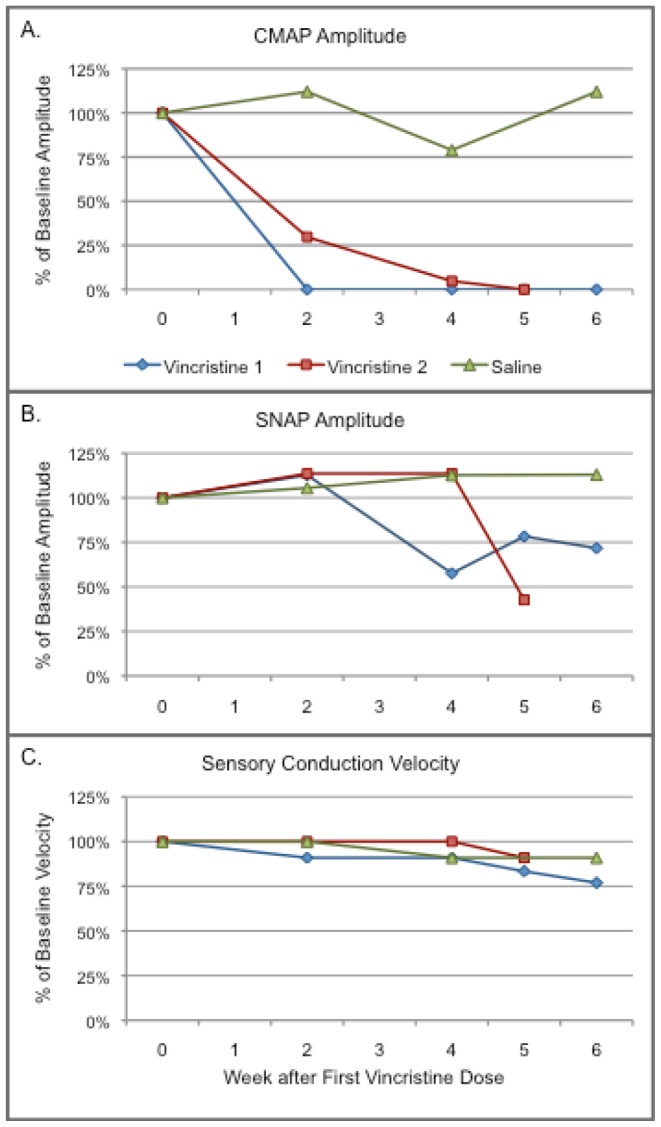
Graphs of repeated measures before and after vincristine. A. CMAP amplitudes, B. SNAP amplitudes and C. sensory conduction velocities. Two rabbits received vincristine (blue line with diamond markers and red line with square markers) and one received saline (green line with triangle markers). Vincristine reduced the SNAP amplitude moderately at a later time than severe CMAP attenuation. While the low number of rabbits precluded meaningful statistical analysis, the results do suggest that evaluating the time course of a peripheral neuropathy with repeated measures is viable in the rabbit.

Two of the three available rabbits received weekly intravenous vincristine while the third rabbit received weekly intravenous saline. Both vincristine rabbits showed an early, severe attenuation of the CMAP amplitude (Fig. 3). Both developed distal weakness in four limbs that evolved over days after the CMAPs were no longer detected. Vincristine also reduced the peroneal SNAP amplitude to a much greater extent than the test-retest variability seen in the 6 healthy rabbits (Fig. 3, Table 1). SNAP amplitude reduction began after CMAPs were no longer identified. Motor conduction velocity did not change or could not be measured. Motor distal latency increased from 2.8 to 4.1 ms in the one rabbit that retained a CMAP 4 weeks after the first vincristine dose. Sensory conduction velocity was unchanged until dropping 20% from baseline in the final test session in one rabbit (Fig. 3). Sensory latency increased 0.35 ms in the same rabbit over the same timeframe. One vincristine rabbit developed foot edema that prevented measurements in the final test session.

In the rabbit that received saline, the peroneal CMAP and SNAP amplitudes and sensorimotor conduction velocities varied by a magnitude similar to the test-retest variability in healthy rabbits (Fig. 3, Table 1).

## Discussion

Noninvasive peroneal nerve motor and sensory studies are feasible in the rabbit distal hindlimb. The rabbit CMAP and SNAP waveforms are similar to humans though the CMAP amplitude is smaller than recordings from humans [18]. The medial and lateral anatomic separation of the deep and superficial peroneal nerves facilitated supramaximal stimulation of a distal cutaneous nerve SNAP uncontaminated by a CMAP (Fig.1).

The coefficient of variation provided a measure of the intertest variability of the nerve conduction measures. Neuromuscular measurements often have coefficient of variations of about 10–15% or less [19]–[21]. All of the rabbit measures fell within this range except for the CMAP amplitude, which had a coefficient of variation closer to 20%. The greater intertest variability of the motor potential amplitudes compared to SNAP amplitudes may be due to the narrow configuration of the extensor digitorum brevis, while the superficial peroneal nerve has a broad fan shape (Fig 1).

The rabbit peroneal nerve sensorimotor recordings measured the time course of a peripheral nerve disorder induced by the neurotoxin vincristine. Intravenous vincristine abolished the CMAP, followed by a reduction of the peroneal SNAP amplitude. The motor predominance of vincristine neuropathy in this study is similar to reports of vincristine neuropathy in children [15], [22], [23]. While vincristine neuropathy in human adults typically presents with sensory symptoms, especially paresthesias, prominent changes in motor strength and CMAP amplitude can also occur in the adult [14], [23].

The rabbits used in this study had been previously studied [16]. The possibility of residual effects and the small number of animals preclude conclusions about vincristine neuropathy in these rabbits beyond demonstrating the methodology's potential for quantifying peripheral nerve disorders.

The surface electrode recordings in this study measured the evoked responses of large, myelinated sensory and motor nerve fibers. The ability to elicit the rabbit SNAP from a distal cutaneous nerve may be particularly valuable in assessing animal models of large-fiber sensory neuropathies. The nerve conduction methods do not detect the responses of small unmyelinated or thinly myelinated cutaneous nerve fibers, which may be selectively damaged in small fiber neuropathies [24]. Small cutaneous nerves, however, can be studied in animals with a skin biopsy and intraepidermal nerve fiber quantification [25], [26].

Rat and mice models are the core, small animal models of peripheral neuropathies and are used for identifying protective therapeutic drugs [27]–[31]. Agents that prevent or contain rodent models of chronic peripheral neuropathy, however, have had difficulty translating into human therapy [30]–[32]. There is indirect evidence supporting the postulate that the probability of neuroprotection in humans may be increased if an agent is protective, not only in rodents, but also in rabbits. Several studies conclude that rabbits (order Lagamorpha) are more closely related to humans on the phylogenetic tree than are rats or mice (order Rodentia) [33], [34]. Rabbits and rodents have different potential for modeling specific human diseases including Alzheimer's disease, hyperlipidemia, maternal diabetes and cystic fibrosis [35]–[38]. The relevance of these genetic studies and animal models to peripheral nerve disorders, however, is unclear.

Rodent sensorimotor nerve conduction studies, performed on the tail and hindlimb with needle electrodes, can measure the time course of a toxin-induced neuropathy [39], [40]. A direct comparison between the rabbit methods with surface nerve recordings and the widely employed rodent procedures has not been made. Therefore comparative statements require caution.

An advantage of the rabbit methods is the ability to record distal sensory potentials that have amplitudes, configurations and recording techniques that are similar to humans, facilitating electrophysiological comparison between animal models and human disorders. The rabbit’s prominent sensory potentials have satisfactory reproducibility and appear suitable for measuring the sensory nerve refractory period and perhaps other markers of sensory nerve excitability [41], [42]. The rabbit nerve conduction studies are not technically demanding for an investigator experienced with either investigative electrophysiology or clinical electromyography. The rabbit distal sensory potential may be valuable for measuring large sensory fiber function in chronic models of neuropathies.
